# Inter-Variability Study of COVLIAS 1.0: Hybrid Deep Learning Models for COVID-19 Lung Segmentation in Computed Tomography

**DOI:** 10.3390/diagnostics11112025

**Published:** 2021-11-01

**Authors:** Jasjit S. Suri, Sushant Agarwal, Pranav Elavarthi, Rajesh Pathak, Vedmanvitha Ketireddy, Marta Columbu, Luca Saba, Suneet K. Gupta, Gavino Faa, Inder M. Singh, Monika Turk, Paramjit S. Chadha, Amer M. Johri, Narendra N. Khanna, Klaudija Viskovic, Sophie Mavrogeni, John R. Laird, Gyan Pareek, Martin Miner, David W. Sobel, Antonella Balestrieri, Petros P. Sfikakis, George Tsoulfas, Athanasios Protogerou, Durga Prasanna Misra, Vikas Agarwal, George D. Kitas, Jagjit S. Teji, Mustafa Al-Maini, Surinder K. Dhanjil, Andrew Nicolaides, Aditya Sharma, Vijay Rathore, Mostafa Fatemi, Azra Alizad, Pudukode R. Krishnan, Nagy Ferenc, Zoltan Ruzsa, Archna Gupta, Subbaram Naidu, Mannudeep K. Kalra

**Affiliations:** 1 Stroke Diagnostic and Monitoring Division, AtheroPoint™, Roseville, CA 95661, USA; drindersingh1@gmail.com (I.M.S.); pomchadha@gmail.com (P.S.C.); 2 Advanced Knowledge Engineering Centre, GBTI, Roseville, CA 95661, USA; sushant.ag09@gmail.com (S.A.); pmelavarthi@gmail.com (P.E.); 3 Department of Computer Science Engineering, PSIT, Kanpur 209305, India; 4 Thomas Jefferson High School for Science and Technology, Alexandria, VA 22312, USA; 5 Department of Computer Science Engineering, Rawatpura Sarkar University, Raipur 492001, India; drrkpathak20@gmail.com; 6 Mira Loma High School, Sacramento, CA 95821, USA; manvi.ketireddy@gmail.com; 7 Department of Radiology, Azienda Ospedaliero Universitaria (A.O.U.), 10015 Cagliari, Italy; martagiuliacol@gmail.com (M.C.); lucasabamd@gmail.com (L.S.); antonellabalestrieri@hotmail.com (A.B.); 8 Department of Computer Science, Bennett University, Noida 201310, India; suneet.gupta@bennett.edu.in; 9 Department of Pathology, Azienda Ospedaliero Universitaria (A.O.U.), 10015 Cagliari, Italy; gavinofaa@gmail.com; 10 The Hanse-Wissenschaftskolleg Institute for Advanced Study, 27753 Delmenhorst, Germany; monika.turk84@gmail.com; 11 Department of Medicine, Division of Cardiology, Queen’s University, Kingston, ON K7L 3N6, Canada; johria@queensu.ca; 12 Department of Cardiology, Indraprastha APOLLO Hospitals, New Delhi 110076, India; drnnkhanna@gmail.com; 13 University Hospital for Infectious Diseases, 10000 Zagreb, Croatia; klaudija.viskovic@bfm.hr; 14 Cardiology Clinic, Onassis Cardiac Surgery Center, 10558 Athens, Greece; soma13@otenet.gr; 15 Heart and Vascular Institute, Adventist Health St. Helena, St. Helena, CA 94574, USA; Lairdjr@ah.org; 16 Minimally Invasive Urology Institute, Brown University, Providence, RI 02912, USA; gyan_pareek@brown.edu (G.P.); dwsobel@gmail.com (D.W.S.); 17 Men’s Health Center, Miriam Hospital, Providence, RI 02906, USA; martin_miner@brown.edu; 18 Rheumatology Unit, National & Kapodistrian University of Athens, 10679 Athens, Greece; psfikakis@med.uoa.gr; 19 Aristoteleion University of Thessaloniki, 54636 Thessaloniki, Greece; tsoulfasg@gmail.com; 20 National & Kapodistrian University of Athens, 10679 Athens, Greece; aprotog@med.uoa.gr; 21 Department of Immunology, Sanjay Gandhi Postgraduate Institute of Medical Sciences, Lucknow 226014, India; durgapmisra@gmail.com (D.P.M.); vikasagr@yahoo.com (V.A.); 22 Academic Affairs, Dudley Group NHS Foundation Trust, Dudley DY1 2HQ, UK; george.kitas@nhs.net; 23 Arthritis Research UK Epidemiology Unit, Manchester University, Manchester M13 9PT, UK; 24 Ann and Robert H. Lurie Children’s Hospital of Chicago, Chicago, IL 60611, USA; jteji@mercy-chicago.org; 25 Allergy, Clinical Immunology and Rheumatology Institute, Toronto, ON L4Z 4C4, Canada; almaini@hotmail.com; 26 AtheroPoint LLC, Roseville, CA 95611, USA; surinderdhanjil@gmail.com (S.K.D.); Vijay.s.rathore@kp.org (V.R.); 27 Vascular Screening and Diagnostic Centre, University of Nicosia Medical School, Nicosia 2368, Cyprus; anicolaides1@gmail.com; 28 Division of Cardiovascular Medicine, University of Virginia, Charlottesville, VA 22904, USA; AS8AH@hscmail.mcc.virginia.edu; 29 Department of Physiology & Biomedical Engineering, Mayo Clinic College of Medicine and Science, Rochester, MN 55905, USA; fatemi.mostafa@mayo.edu; 30 Department of Radiology, Mayo Clinic College of Medicine and Science, Rochester, MN 55905, USA; Alizad.Azra@mayo.edu; 31 Neurology Department, Fortis Hospital, Bangalore 560076, India; prkrish12@rediffmail.com; 32 Internal Medicine Department, University of Szeged, 6725 Szeged, Hungary; drnagytfer@hotmail.com; 33 Zoltan Invasive Cardiology Division, University of Szeged, 6725 Szeged, Hungary; zruzsa@icloud.com; 34 Radiology Department, Sanjay Gandhi Postgraduate Institute of Medical Sciences, Lucknow 226014, India; garchna@gmail.com; 35 Electrical Engineering Department, University of Minnesota, Duluth, MN 55812, USA; dsnaidu@d.umn.edu; 36 Department of Radiology, Massachusetts General Hospital, 55 Fruit Street, Boston, MA 02114, USA; mkalra@mgh.harvard.edu

**Keywords:** COVID-19, computed tomography, lungs, variability, segmentation, hybrid deep learning

## Abstract

*Background*: For COVID-19 lung severity, segmentation of lungs on computed tomography (CT) is the first crucial step. Current deep learning (DL)-based Artificial Intelligence (AI) models have a bias in the training stage of segmentation because only one set of ground truth (GT) annotations are evaluated. We propose a robust and stable inter-variability analysis of CT lung segmentation in COVID-19 to avoid the effect of bias. *Methodology*: The proposed inter-variability study consists of two GT tracers for lung segmentation on chest CT. Three AI models, PSP Net, VGG-SegNet, and ResNet-SegNet, were trained using GT annotations. We hypothesized that if AI models are trained on the GT tracings from multiple experience levels, and if the AI performance on the test data between these AI models is within the 5% range, one can consider such an AI model robust and unbiased. The K5 protocol (training to testing: 80%:20%) was adapted. Ten kinds of metrics were used for performance evaluation. *Results*: The database consisted of 5000 CT chest images from 72 COVID-19-infected patients. By computing the coefficient of correlations (CC) between the output of the two AI models trained corresponding to the two GT tracers, computing their differences in their CC, and repeating the process for all three AI-models, we show the differences as 0%, 0.51%, and 2.04% (all < 5%), thereby validating the hypothesis. The performance was comparable; however, it had the following order: ResNet-SegNet > PSP Net > VGG-SegNet. *Conclusions*: The AI models were clinically robust and stable during the inter-variability analysis on the CT lung segmentation on COVID-19 patients.

## 1. Introduction

The WHO’s International Health Regulations and Emergency Committee (IHREC) proclaimed COVID-19 a “public health emergency of international significance” or “pandemic” on 30 January 2020. More than 231 million people have been infected worldwide, and nearly 4.7 million people have died due to COVID-19 [1]. Although this “severe acute respiratory syndrome coronavirus 2” (SARS-CoV-2) virus specifically targets the pulmonary and vascular system, it has the potential to travel through the body and lead to complications such as pulmonary embolism [2], myocardial infarction, stroke, or mesenteric ischemia [3,4,5]. Comorbidities such as diabetes mellitus, hypertension, and obesity substantially increase the severity and mortality of COVID-19 [6,7]. A real-time reverse transcription-polymerase chain reaction (RT-PCR) is the recommended method for diagnosis [8]. Chest radiographs and computed tomography (CT) [9,10,11] are used to determine disease severity in patients with moderate to severe disease or underlying comorbidities based on the extent of pulmonary opacities such as ground-glass (GGO), consolidation, and mixed opacities in CT scans [7,12,13,14].

Most radiologists provide a semantic description of the extent and type of opacities to describe the severity of COVID-19 pneumonia. The semiquantitative evaluation of pulmonary opacities is time-consuming, subjective, and tedious [15,16,17,18]. Thus, there is a need for a fast and error-free early COVID-19 disease diagnosis and real-time prognosis solutions. Machine learning (ML) offers a solution to this problem by providing a rich set of algorithms [19]. Previously, ML has been used for detection of cancers in breast [20], liver [21,22], thyroid [23,24,25], skin [26,27], prostate [28,29], ovary [30], and lung [31]. There are two main components in disease detection, i.e., segmentation [32,33,34,35] and classification [36,37], where segmentation plays a crucial step. An extension of ML called deep learning (DL) employs dense layers to automatically extract and classify all relevant imaging features [38,39,40,41,42,43]. Hybrid DL (HDL), a method that combines two DL systems, helps address some of the challenges in solo DL models [44,45]. This includes overfitting and optimization of hyperparameters, thereby removing the bias [45].

During the AI model training, the most crucial stage is the ground truth (GT) annotation of organs that need to be segmented. It is a time-consuming operation with monetary constraints since skilled personnel such as radiologists are expensive to recruit and difficult to find. These annotations, if conducted by one tracer, make the AI system biased. A plurality of tracers being used to produce the GT annotated dataset makes the system more resilient and lowers the AI bias [46,47,48,49]. This is because the AI model can grasp and adjust to the sensitivity of the difference in the tracings of the tracers. Thus, to avoid AI bias, one needs to have an automated AI-based system with multiple tracers. To establish the validity of such automated AI systems, one must undergo inter-variability analysis with two or more observers.

**Figure 1:**
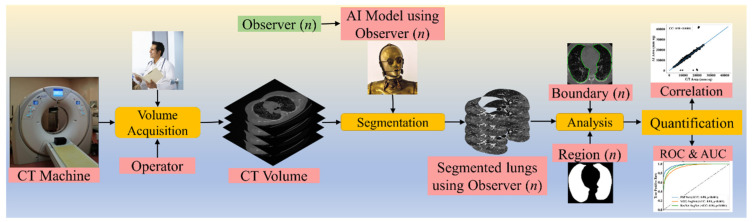
COVLIAS 1.0: Inter-variability analysis of CT-based lung segmentation and quantification system for COVID-19 patients. ROC: Receiver operating characteristic; AUC: Area-under-the-curve.

To validate the AI systems, we hypothesize that two conditions must be met: (a) the two observers should perform within 5% range of each other and (b) the performance of the AI system using the ground truth tracings from these two observers should also be within the 5% threshold [48]. The AI performance is computed between the GT-area and the AI model-estimated area. The focus of the proposed research is to design a reliable AI system based on the inter-observer paradigm. Figure 1 depicts a COVID-19 CT lung segmentation system in which the CT machine is used to acquire CT volumes. This volume is then annotated by multiple observers (Figure 1, *n* denotes the number of observers), and multiple AI models are generated, which is then used for lung segmentation. The segmentation output is the binary mask of the lung, its boundary, and the corresponding boundary overlays. This output can be used for evaluating the performance, analysis, and quantification of the results.

The layout of this inter-variability study is as follows: Section 2 presents the methodology with the demographics, COVLIAS 1.0 pipeline, AI architectures, and loss functions. The experimental protocol is shown in Section 3, while results and performance evaluation are presented in Section 4. The discussions and conclusions are presented in Section 5 and Section 6, respectively.

## 2. Methodology

### 2.1. Patient Demographics, Image Acquisition, and Data Preparation

#### 2.1.1. Demographics

The dataset consists of 72 adult Italian patients with 46 being male and the remaining being female. The mean height and weight were 173 cm and 79 kg, respectively. A total of 60 patients tested positive for RT-PCR, while 12 patients were confirmed using broncho-alveolar lavage [50]. Overall, the cohort had an average of 4.1 GGO, which was considered low.

#### 2.1.2. Image Acquisition

**Figure 2:**
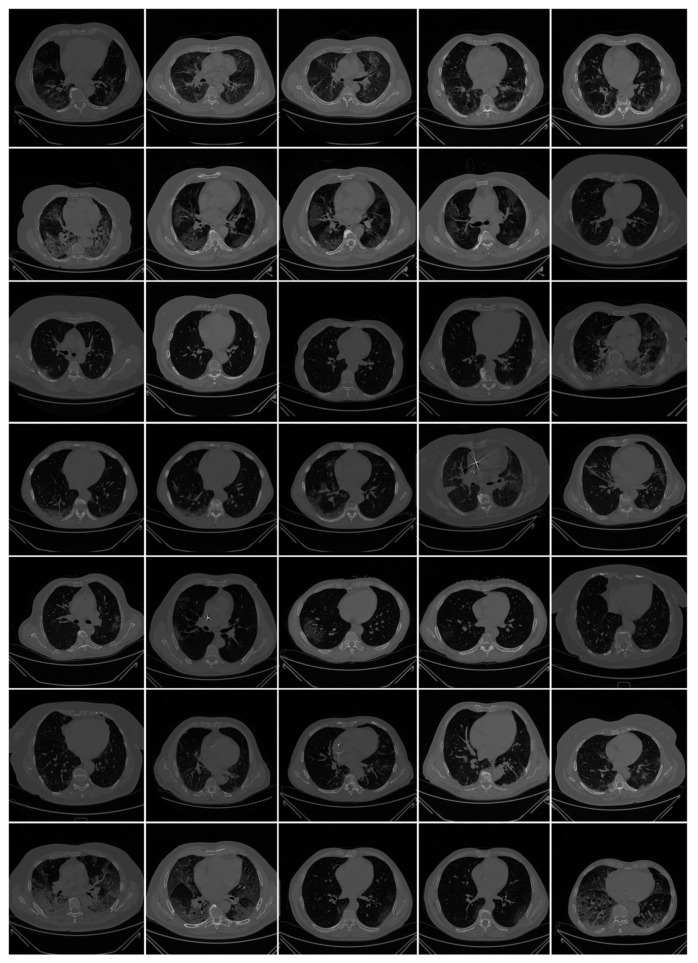
Raw lung COVID-19 CT scans taken from different patients in the database.

All chest CT scans were performed in a supine posture during a single full inspiratory breath-hold using a 128-slice multidetector-row Philips Healthcare’s “Philips Ingenuity Core” CT scanner. There were no intravenous or oral contrast media administrations. The CT exams were performed using a 120 kV, 226 mAs/slice (utilizing an automatic tube current modulation—Z-DOM by Philips), a 1.08 spiral pitch factor, 0.5-s gantry rotation time, and 64 × 0.625 detector setup. Soft tissue kernel with 512 × 512 matrix (mediastinal window) and lung kernel with 768 × 768 matrix (lung window) was used to reconstruct 1 mm-thick images. The Picture Archiving and Communication System (PACS) workstation that was utilized to review the CT images was outfitted with two Eizo 35 × 43 cm displays with a 2048 × 1536 matrix. Figure 2 shows the raw sample CT scans of COVID-19 patients with varying lung sizes and variable intensity patterns, posing a challenge.

#### 2.1.3. Data Preparation

**Figure 3:**
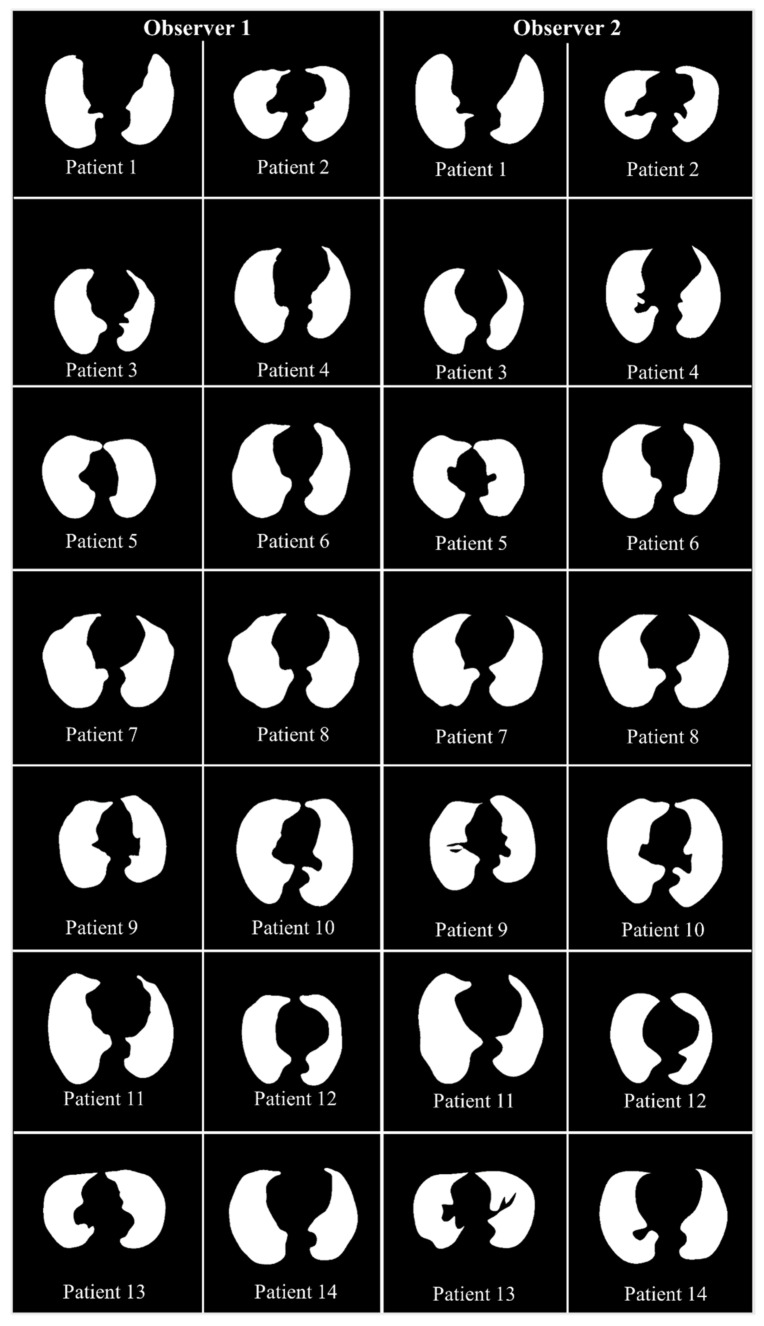
GT white binary mask for AI model training for Observer 1 vs. Observer 2.

The proposed study makes use of the CT data of 72 COVID-positive individuals. Each patient had 200 slices, out of which the radiologist [LS] chose 65–70 slices from the visible lung region, resulting in 5000 images in total. The AI-based segmentation models were trained and tested using these 5000 images. To prepare the data for segmentation, a binary mask was created manually in a selected slice with the help of ImgTracer™ under the supervision of a qualified radiologist [LS] (Global Biomedical Technologies, Inc., Roseville, CA, USA) [47,48,51]. Figure 3 shows the white binary mask of the lung region computed using ImgTracer™ during manual tracings by Observer 1 and 2 (both were postgraduate researchers trained by our radiological team).

### 2.2. Architecture

COVLIAS 1.0 system incorporates three models: one solo DL (SDL) and two hybrid DL (HDL). The proposed study incorporates three AI models: (a) PSP Net, (b) VGG-SegNet, and (c) ResNet-SegNet.

#### 2.2.1. Three AI Models: PSP Net, VGG-SegNet, and ResNet-SegNet

**Figure 4:**
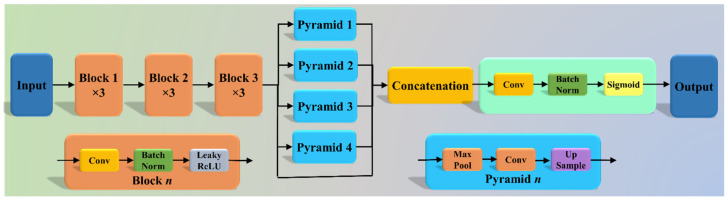
PSP Net architecture.

The Pyramid Scene Parsing Network (PSP Net) [52] is a semantic segmentation network with the ability to consider the global context of the image. The architecture of PSP Net (Figure 4) has four parts: (i) input, (ii) feature map, (iii) pyramid pooling module, and (iv) output. The input to the network is the image to be segmented, which undergoes extraction of the feature map using a set of dilated convolution and pooling blocks. The dilated convolution layer is added at the last two blocks of the network to keep more prominent features at the end. The next stage is the pyramid pooling module; it is the heart of the network, as it helps capture the global context of the image/feature map generated in the previous step. This section consists of four parts, each with a different scaling ability. The scaling of this module includes 1, 2, 3, and 6, where 1 × 1 scaling helps capture the spatial features and thereby increases the resolution of the features captured. The 6 × 6 scaling captures the higher-resolution features. At the end of this module, all the output from these four parts is pooled using global average pooling. For the last part, the global average pooling output is fed to a set of convolutional layers. Finally, the set of prediction classes are generated as the output binary mask.

**Figure 5:**
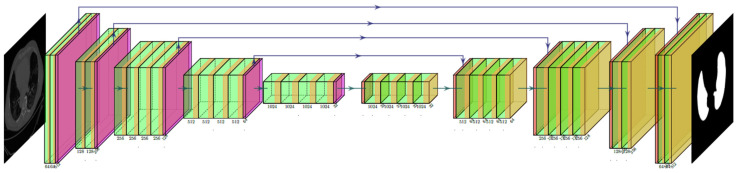
VGG-SegNet architecture.

The VGGNet architecture (Figure 5) was designed to reduce the training time by replacing the kernel filter in the initial layer with an 11 and 5 sized filter, thereby reducing the # of parameters in the two-dimension convolution (Conv) layers [53]. The VGG-SegNet architecture used in this study is composed of three parts (i) encoder, (ii) decoder part, and (iii) a pixel-wise SoftMax classifier at the end. It consists of 16 Conv layers compared to the SegNet architecture, where only 13 Conv layers are used [54] in the encoder part. This increase in #layers helps the model extract more features from the image. The final output of the model is a binary mask with the lung region annotated as 1 (white) and the rest of the image as 0 (black).

**Figure 6:**
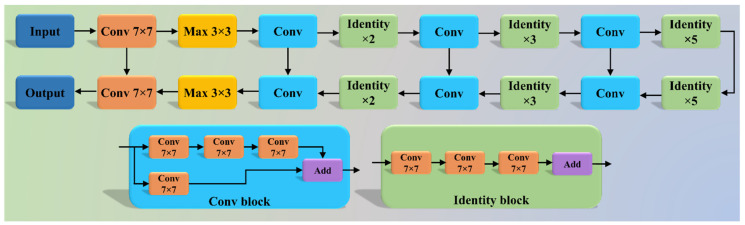
ResNet-SegNet architecture.

Although VGGNet was very efficient and fast, it suffered from the problem of vanishing gradients. It results in significantly less or no weight training during backpropagation; at each epoch, it keeps getting multiplied with the gradient, and the update to the initial layers is very small. To overcome this problem, Residual Network or ResNet [55] came into existence (Figure 6). In this architecture, a new connection was introduced known as skip connection which allowed the gradients to bypass a certain number of layers, solving the vanishing gradient problem. Moreover, with the help of one more additions to the network, i.e., an identity function, the local gradient value was kept to one during the backpropagation step.

#### 2.2.2. Loss Functions for AI Models

The proposed system uses cross-entropy (CE)-loss during the training of the AI models. Equation (1) below represents the CE-loss, symbolized as $l_{CE}$, for the three AI models:

(1) $$l_{CE}=-[(x_{i}{\;\times \;\log\;p}_{i})\;+\;(1\;-\;x_{i}{)\;\times \;\log(1\;-\;p}_{i})]$$

where x_i_ represents the input GT label 1, (1 − x_i_) represents the GT label 0, $p_{i}$ represents the probability of the classifier (SoftMax) used at the last layer of the AI model, and × represents the product of the two terms. Figure 4, Figure 5 and Figure 6 presents the three AI architectures that have been trained using the CE-loss function.

## 3. Experimental Protocol

### 3.1. Accuracy Estimation of AI Models Using Cross-Validation

A standardized cross-validation (CV) protocol was adapted for determining the accuracy of the AI models. Our group has published several CV-based protocols of different kinds using AI framework [27,30,37,56,57]. Since the data were moderate, the K5 protocol was used, which consisted of 80% training data (4000 CT images) and 20% testing (1000 CT images). Five folds were designed in such a way that each fold got a chance to have a unique test set. An internal validation mechanism was part of the K5 protocol where 10% data was considered for validation.

### 3.2. Lung Quantification

There were two methods used for quantification of the segmented lungs using AI models. The spirit of these two methods originates from the shape analysis concept. In the first method, lung area (LA) is computed since the region is balloon-shaped, thus the area parameter is well suited for the measurement [58,59]. In the second method, we compute the long-axis of the lung (LLA) since the shape of the lung is more longitudinal than circular. A similar approach was taken for the long-axis view in heart computation [60]. The lung area (LA) was calculated by counting the number of white pixels in the binary mask segmented lungs, and the lung long axis (LLA) was calculated by the most distant distance segment joining anterior to posterior of the lungs. A resolution factor of 0.52 was used to convert (i) pixel to mm^2^ for the LA and (ii) pixel to mm for the LLA computation and quantification.

If the total number of the image is represented by *N* in the database, $A_{ai}\left(m,n\right)$ represents lung area for in the image “*n*” using the AI model “*m*”, ${\bar{A}}_{ai}\left(m\right)$ represents the mean lung area corresponding to the AI model “m,” and mean area of the GT binary mask is represented by ${\bar{A}}_{gt}$, then mathematically ${\bar{A}}_{ai}\left(m\right)$ and ${\bar{A}}_{gt}$ can be computed as shown in Equation (2).

(2) $$\begin{array}{c} {\bar{A}}_{ai}\left(m\right)=\frac{\sum_{n\;=\;1}^{N}A_{ai}\left(m,n\right)}{N}\\ {\bar{A}}_{gt}=\frac{\sum_{n\;=\;1}^{N}A_{gt}\left(n\right)}{N} \end{array}\}$$

Similarly, $LA_{ai}\left(m,n\right)$ represents LLA for in the image “*n*” using the AI model “*m*”, ${\bar{LA}}_{ai}\left(m\right)$ represents the mean LLA corresponding to the AI model “m,”${\bar{LA}}_{gt}$ represents the corresponding mean LLA of the GT binary lung mask, then mathematically ${\bar{LA}}_{ai}\left(m\right)$ and ${\bar{LA}}_{gt}$ can be computed as shown in Equation (3).

(3) $$\begin{array}{c} {\bar{LA}}_{ai}\left(m\right)=\frac{\sum_{n\;=\;1}^{N}LA_{ai}\left(m,n\right)}{N}\\ {\bar{LA}}_{gt}=\frac{\sum_{n\;=\;1}^{N}LA_{gt}\left(n\right)}{N} \end{array}\}$$

### 3.3. AI Model Accuracy Computation

The accuracy of the AI system was measured by comparing the predicted output and the ground truth pixel values. These values were interpreted as binary (0 or 1) numbers as the output lung mask was only black and white, respectively. Finally, these binary numbers were summed up and divided by the total number of pixels in the image. If TP, TN, FN, and FP represent true positive, true negative, false negative, and false positive, then the accuracy of the AI system can be computed as shown in Equation (4) [61].

(4) $$\mathrm{ACC}\;\left(\mathrm{ai}\right)\;\left(\%\right)=\left(\frac{\mathrm{TP}+\mathrm{TN}}{\mathrm{TP}+\mathrm{FN}+\mathrm{TN}+\mathrm{FP}}\right)\times 100$$

## 4. Results and Performance Evaluation

### 4.1. Results

**Figure 7:**
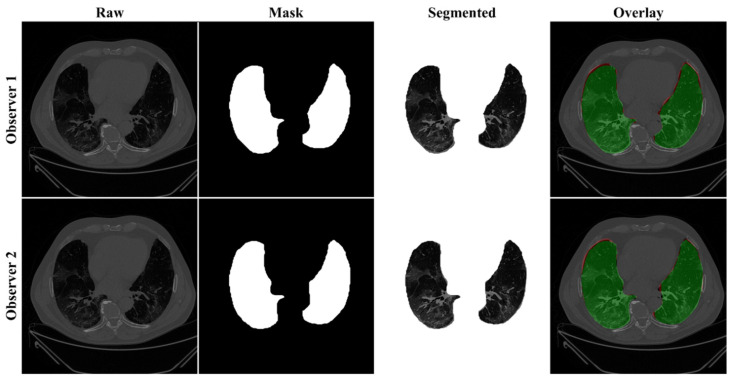
Results from PSP Net while using Observers 1 and 2. Columns are the raw, binary mask output, segmented lung region, and overlay of the estimated lung region vs. ground truth region.

**Figure 8:**
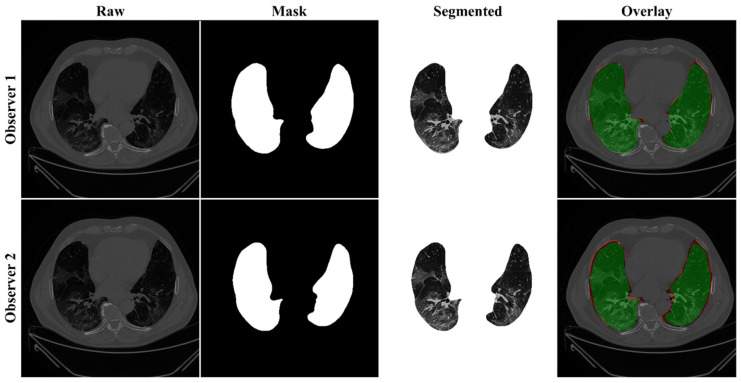
Results from VGG-SegNet while using Observers 1 and 2. Columns are the raw, binary mask output, segmented lung region, and overlay of the estimated lung region vs. ground truth region.

**Figure 9:**
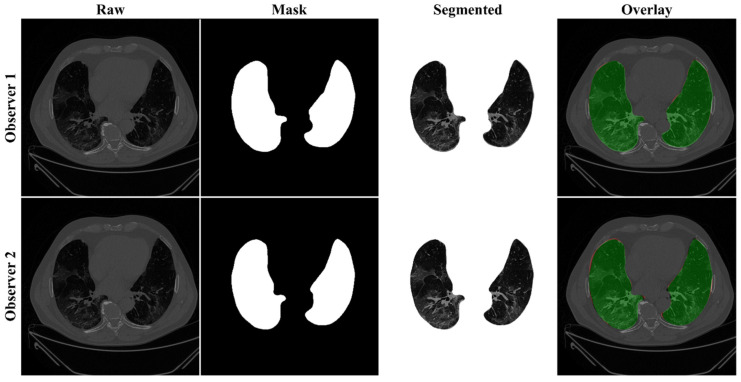
Results from ResNet-SegNet while using Observers 1 and 2. Columns are the raw, binary mask output, segmented lung region, and overlay of the estimated lung region vs. ground truth region.

Previously, COVLIAS 1.0 [54] was designed to run on a training: testing ratio of 2:3 dataset from 5000 images. However, this study proposes an inter-observer variability study with K5 in a CV framework. The training was performed on two sets of annotations, i.e., Observer 1 and Observer 2. The output results are similar to the previously published study, i.e., a binary mask of the segmented lungs. Figure 7, Figure 8 and Figure 9 show the AI-generated binary mask, segmented lung, and color segmented lung with grayscale background as an overlay for the three AI models.

### 4.2. Performance Evaluation

This section deals with the performance evaluation (PE) of the three AI models for Observer 1 vs. Observer 2. Section 4.2.1 presents the visual comparison of the results, which includes (i) boundary overlays against the ground truth boundary and (ii) lung long axis against the ground truth axis. Section 4.2.2 shows the PE for lung area error, which consists of (i) cumulative frequency (CF) plot, (ii) Bland-Altman plot, (iii) Jaccard Index (JI) and Dice Similarity (DS), and (iv) ROC and AUC curves for the three AI-based models’ performance for Observer 1 vs. Observer 2. Similarly, lung long axis error (LLAE) presents PE using (i) cumulative plot, (ii) correlation coefficient (CC), and (iii) Bland-Altman plot. Finally, statistical analyses of the LA and LLA are presented using paired *t*-test, Wilcoxon, Mann-Whitney, and CC values for all 12 possible combinations for three AI models between Observer 1 and Observer 2.

#### 4.2.1. Lung Boundary and Long Axis Visualization

**Figure 10:**
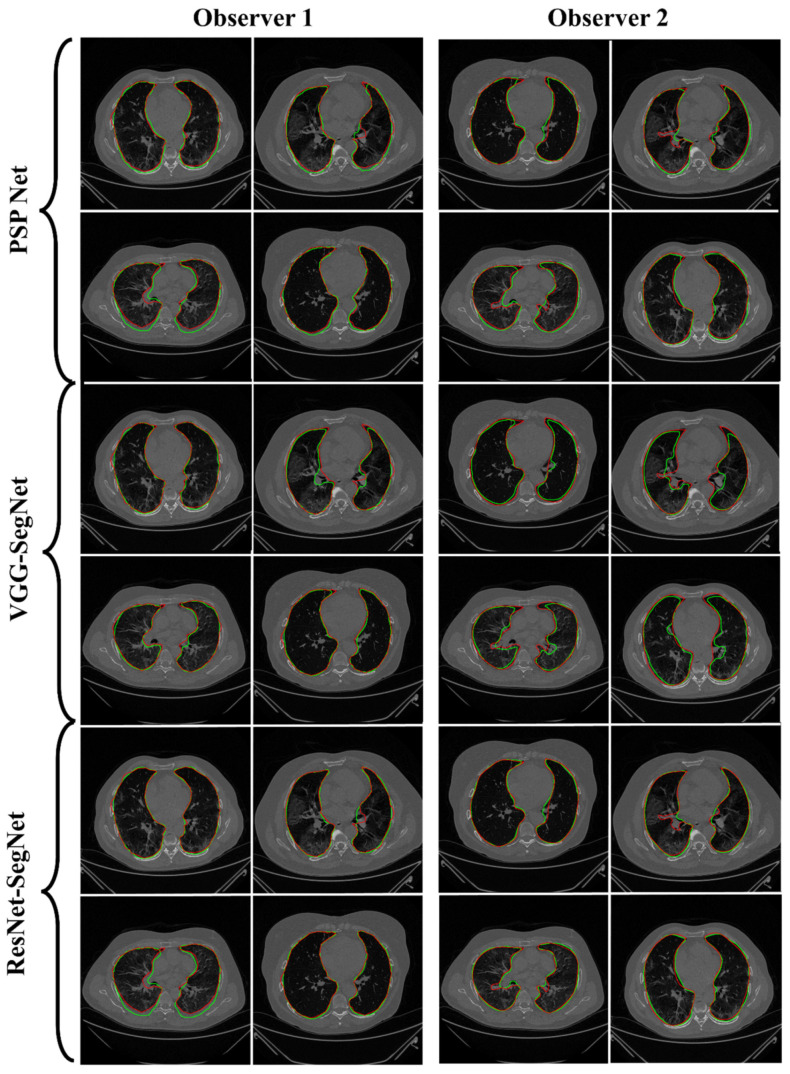
AI-model segmented boundary (green) vs. GT boundary (red) for Observer 1 and Observer 2.

**Figure 11:**
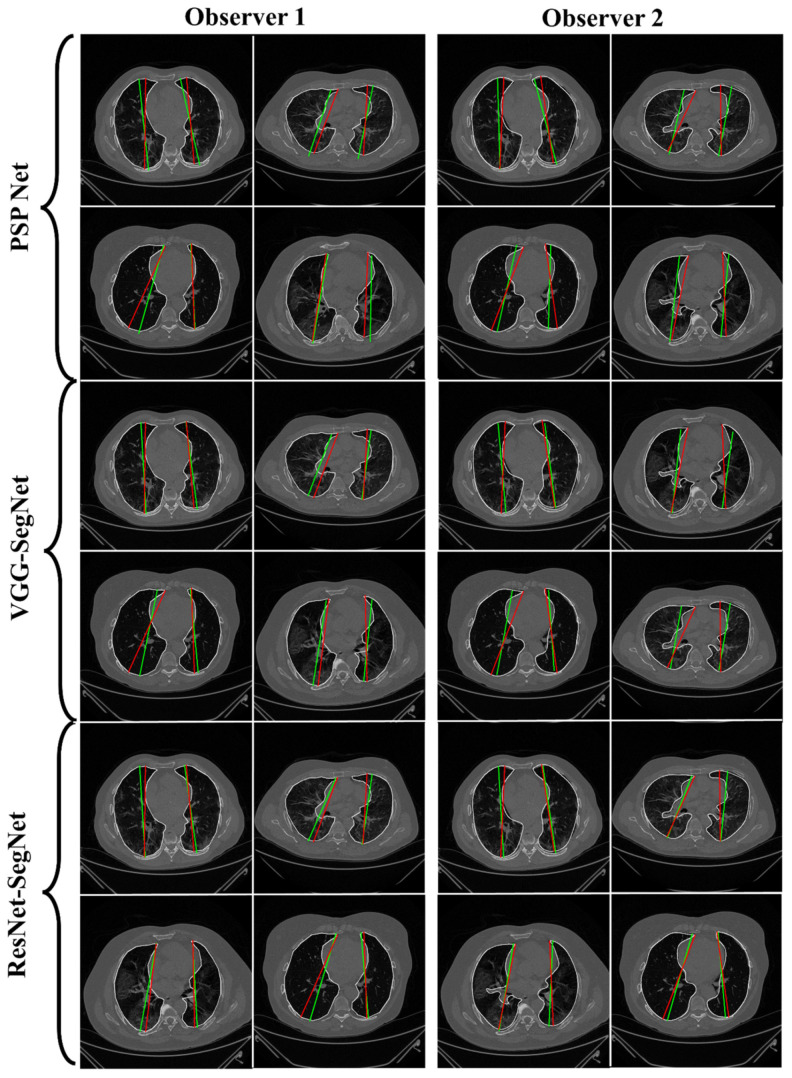
AI-model long axis (green) vs. GT long axis (red) for Observer 1 and Observer 2.

The overlay for the three AI model boundaries (green) and GT-boundary (red) corresponding to Observer 1 (left) and Observer 2 (right) with a grayscale COVID-19 CT slice in the background is shown in Figure 10, while Figure 11 shows the AI-long axis (green) and GT-long axis (red) between Observer 1 and Observer 2 for three AI models. It shows the reach of anterior to posterior of the left and right lungs, with the GT boundary (white) corresponding to Observer 1 (left) and Observer 2 (right) of the lungs by the tracer using ImgTracer™. The three AI models follow the order: PSP Net, VGG-SegNet, and ResNet-SegNet.

#### 4.2.2. Performance Metrics for the Lung Area Error

##### Cumulative Frequency Plot for Lung Area Error

**Figure 12:**
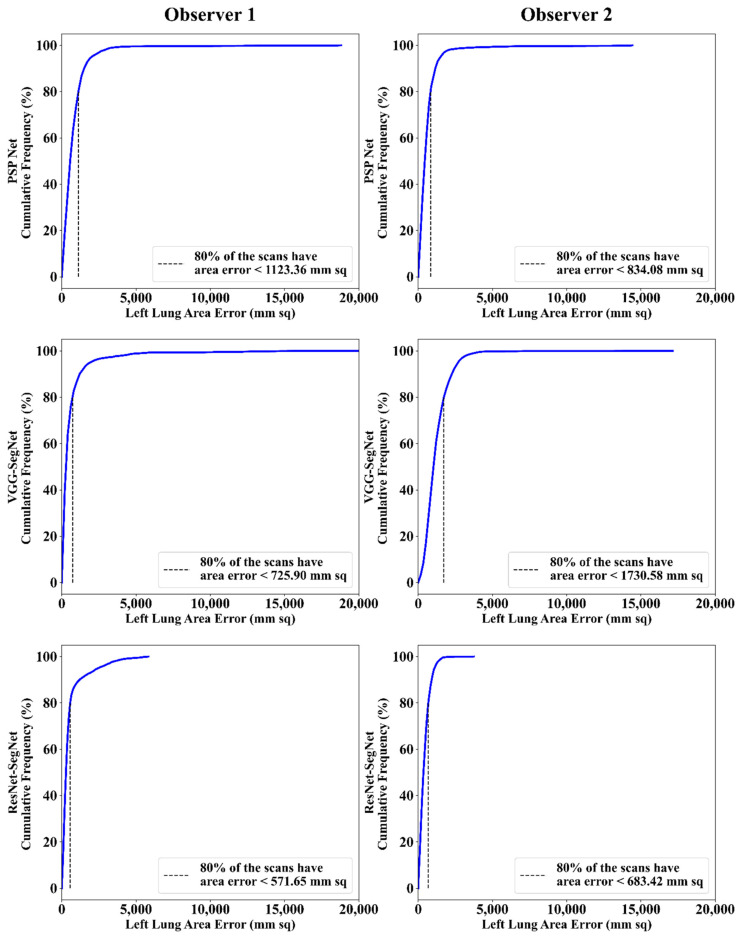
Cumulative frequency plot of left LAE using three AI models: Observer 1 vs. Observer 2.

**Figure 13:**
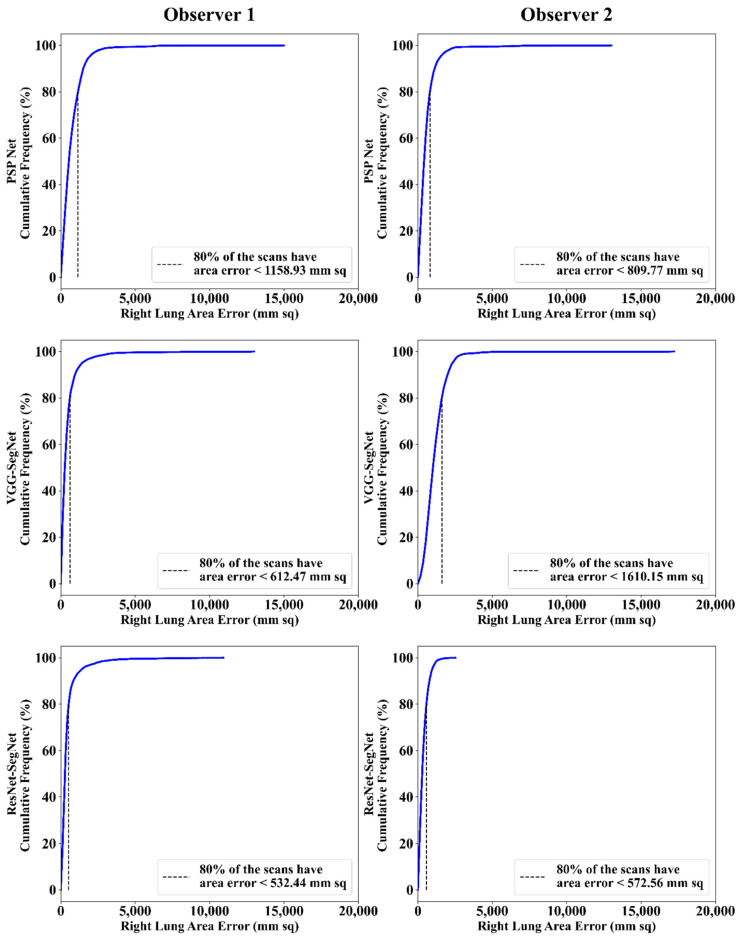
Cumulative frequency plot of right LAE using three AI models: Observer 1 vs. Observer 2.

The frequency of occurrence of the LAE is compared to a reference value in the cumulative frequency analysis and shown in Figure 12 (left lung) and Figure 13 (right lung) for three AI models between Observer 1 and Observer 2. A cutoff-score of 80% was chosen to show the difference between the three AI models. The LAE with the selected cutoff for the left lung was 1123.36 mm^2^, 725.90 mm^2^, and 571.65 mm^2^ for the three AI models using Observer 1, and 834.08 mm^2^, 1730.58 mm^2^, and 683.42 mm^2^, respectively, for the three AI models using Observer 2. A similar trend was followed by the right lung with 1158.93 mm^2^, 612.47 mm^2^, and 532.44 mm^2^ for the three AI models using Observer 1, and 809.77 mm^2^, 1610.15 mm^2^, and 572.56 mm^2^, respectively, for the three AI models using Observer 2. The three AI models follow the order: PSP Net, VGG-SegNet, and ResNet-SegNet.

##### Correlation Plot for Lung Area Error

**Figure 14:**
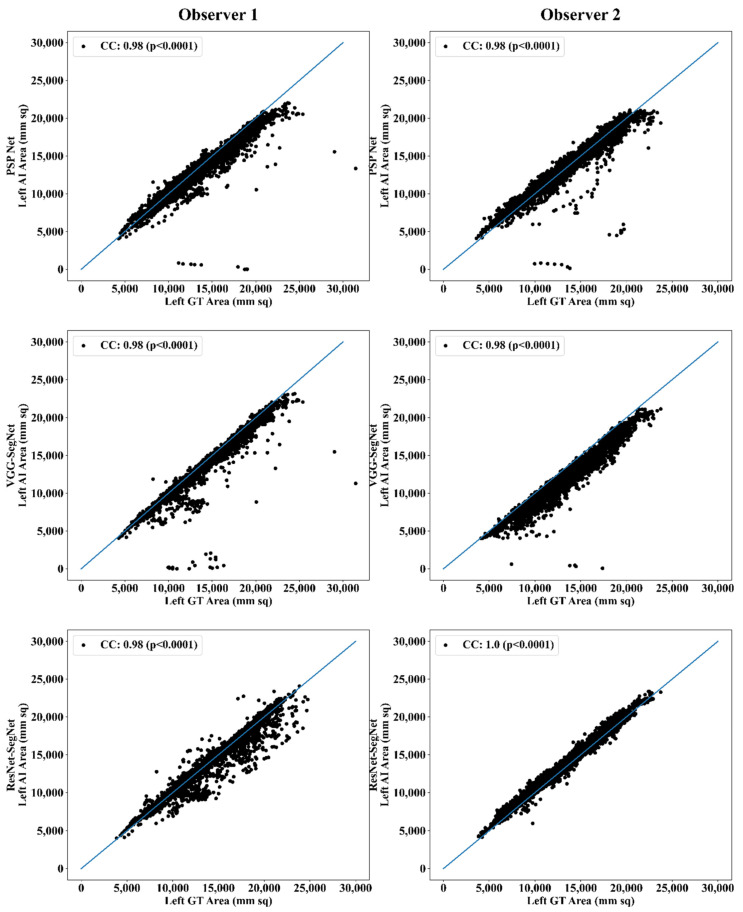
CC of left lung area using three AI models: Observer 1 vs. Observer 2.

**Figure 15:**
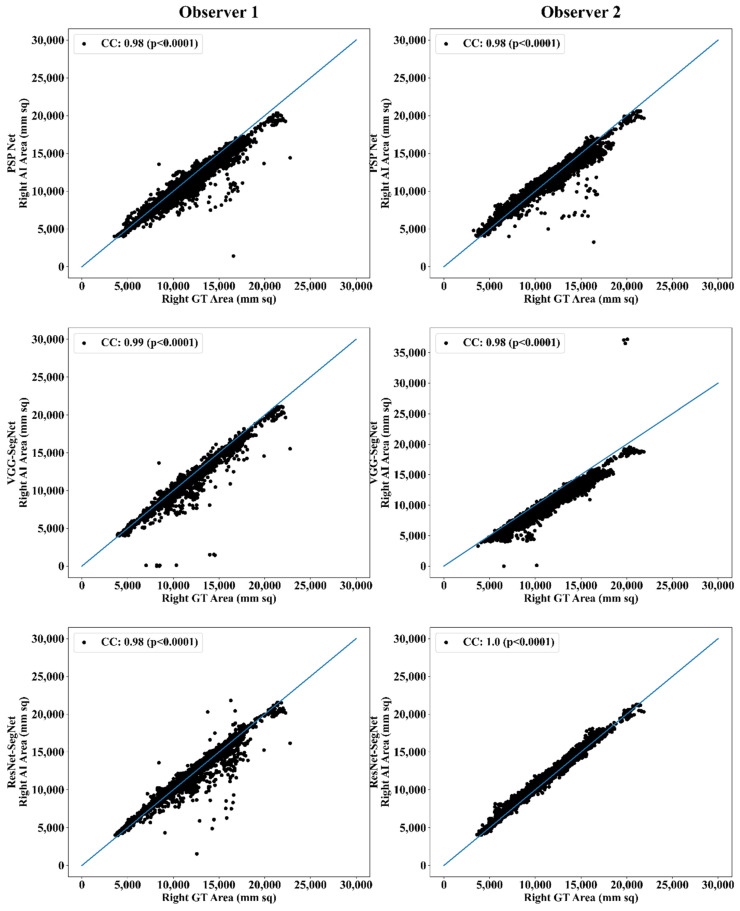
CC of right lung area using three AI models: Observer 1 vs. Observer 2.

Coefficient of correlations (CC) plots for the three AI models’ LA vs. GT, area corresponding to the left and right between Observers 1 and 2, are shown in Figure 14 and Figure 15. The CC values are summarized in Table 1 with a percentage difference between Observers 1 and 2. The percentage difference for the CC value (*p* < 0.001) ranges from 0% to 2.04%, which is <5% as part of the error threshold chosen as the hypothesis. This clearly shows that the AI models are clinically valid for the proposed setting of the inter-observer variability study.

##### Jaccard Index and Dice Similarity

**Figure 16:**
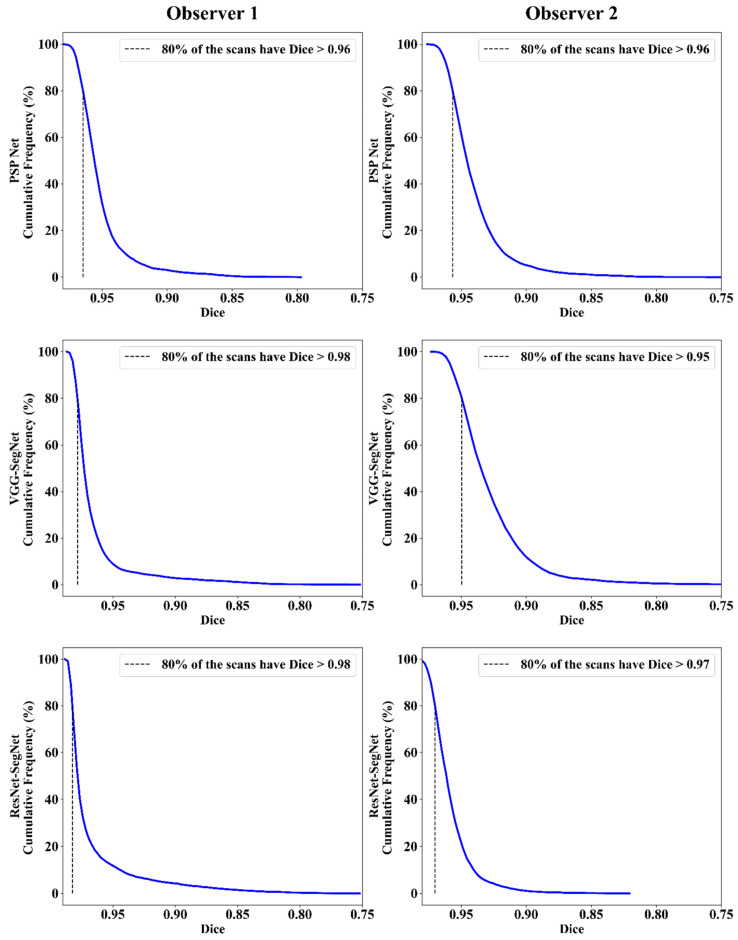
DS for combined lung using the three AI models: Observer 1 vs. Observer 2.

**Figure 17:**
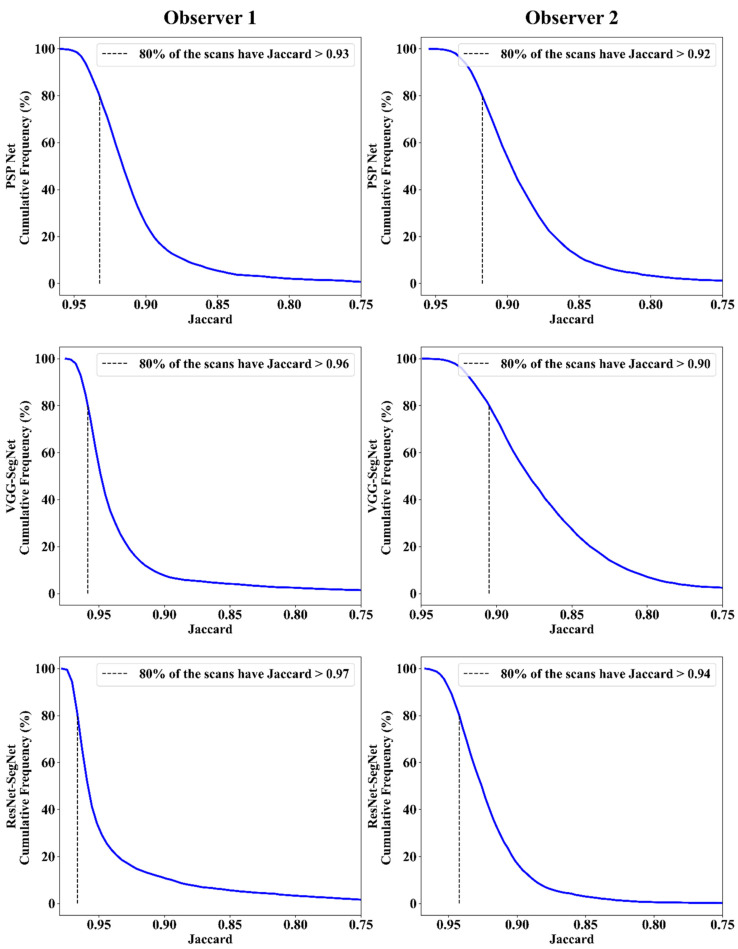
JI for combined lung using three AI models: Observer 1 vs. Observer 2.

Figure 16 depicts a cumulative frequency plot for dice similarity (DS) for three AI models between Observers 1 and Observer 2. It shows that 80% of the CT images had a DS > 0.95. A cumulative frequency plot for the Jaccard Index (JI) is presented in Figure 17 and shows that 80% of the CT scans had a JI > 0.90 between Observer 1 and Observer 2. The three AI models follow the order: PSP Net, VGG-SegNet, and ResNet-SegNet.

##### Bland-Altman Plot for Lung Area

**Figure 18:**
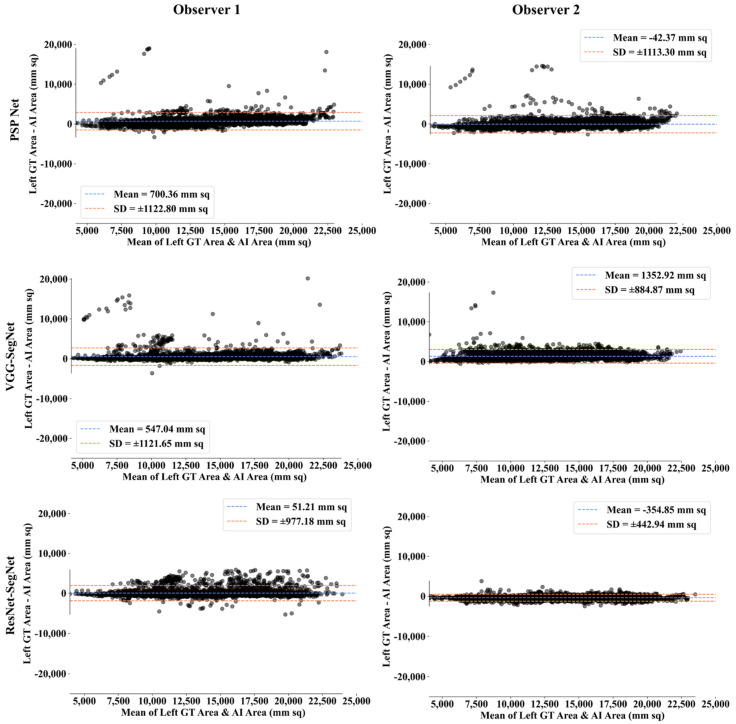
BA for left LA for three AI models: Observer 1 vs. Observer 2.

**Figure 19:**
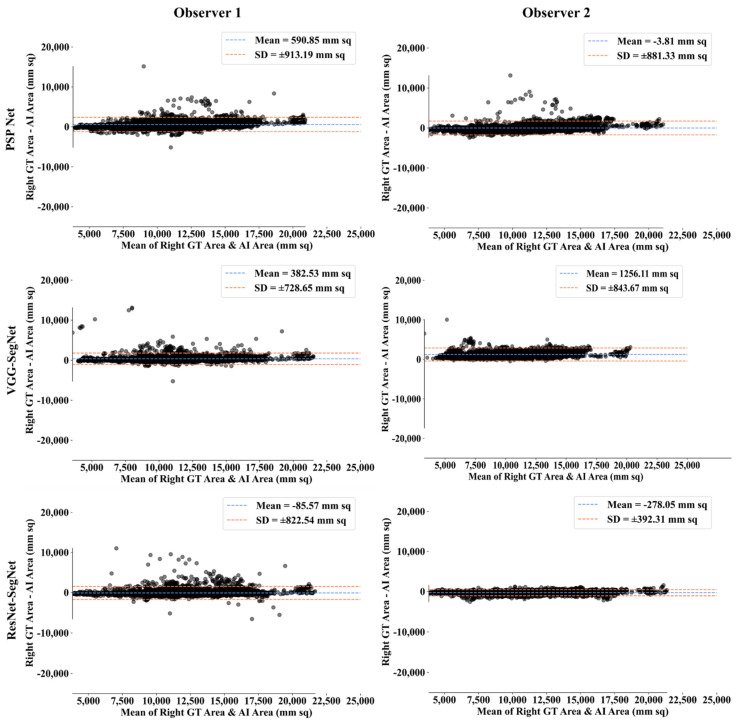
BA for right LA using three AI models: Observer 1 vs. Observer 2.

A Bland-Altman plot is used to demonstrate the consistency of two methods that employ the same variable. Based on our prior paradigms [48,62], we follow the Bland-Altman computing procedure. Figure 18 and Figure 19 show the (i) mean and (ii) standard deviation of the lung area between the AI model and GT area corresponding to Observers 1 and Observer 2.

##### ROC Plots for Lung Area

**Figure 20:**
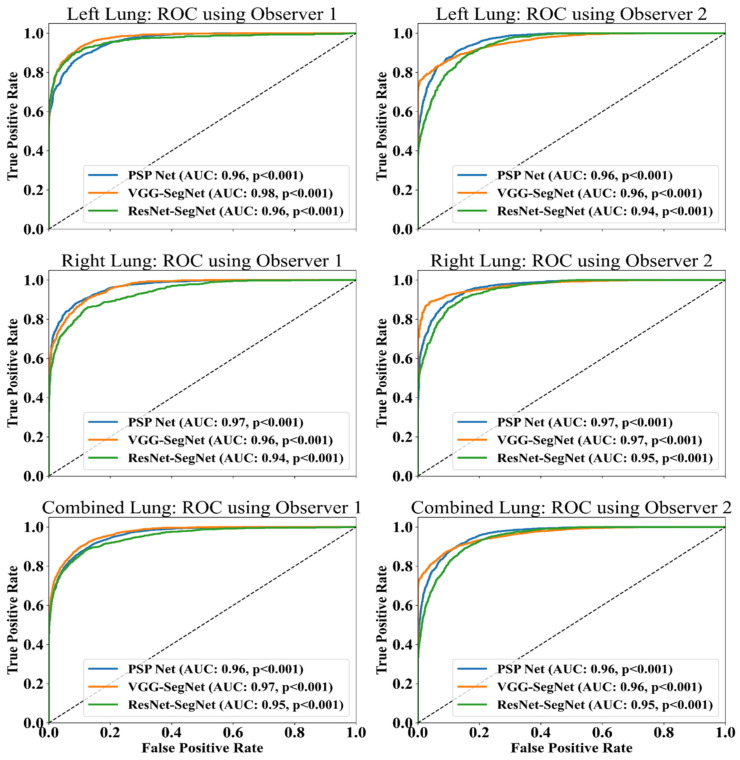
ROC and AUC curve for the three AI models: Observer 1 vs. Observer 2.

An ROC curve represents how an AI system’s diagnostic performance changes as the discrimination threshold changes. Figure 20 shows the ROC curve and corresponding AUC value for the three AI models between Observer 1 and Observer 2. The three AI models follow the order: PSP Net, VGG-SegNet, and ResNet-SegNet.

#### 4.2.3. Performance Evaluation Using Lung Long Axis Error

##### Cumulative Frequency Plot for Lung Long Axis Error

**Figure 21:**
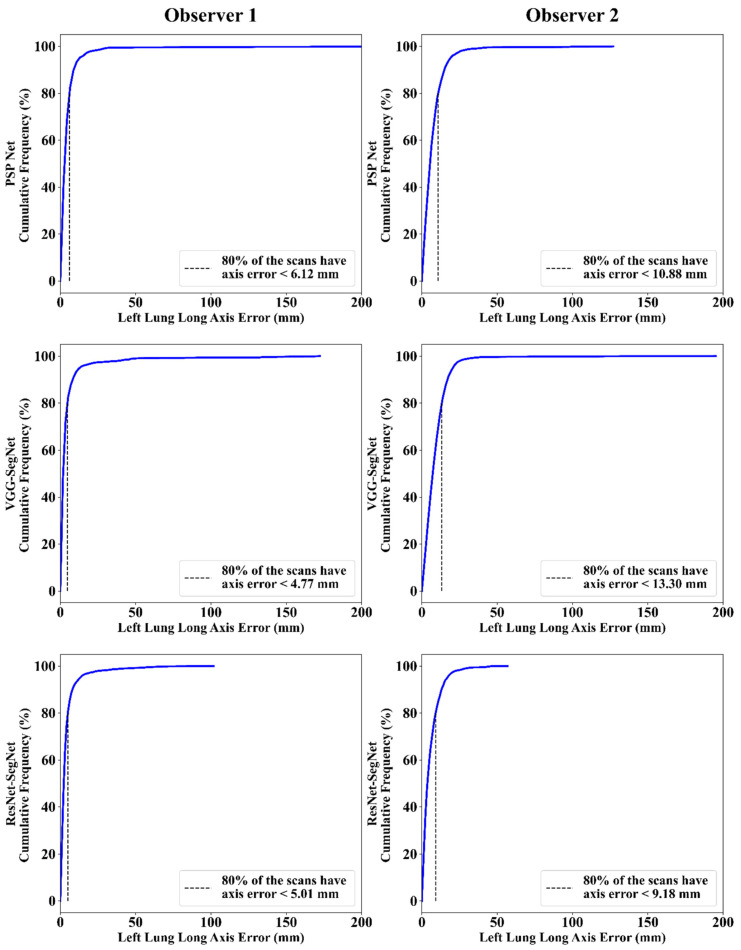
Cumulative frequency plot for left LLAE using three AI models: Observer 1 vs. Observer 2.

**Figure 22:**
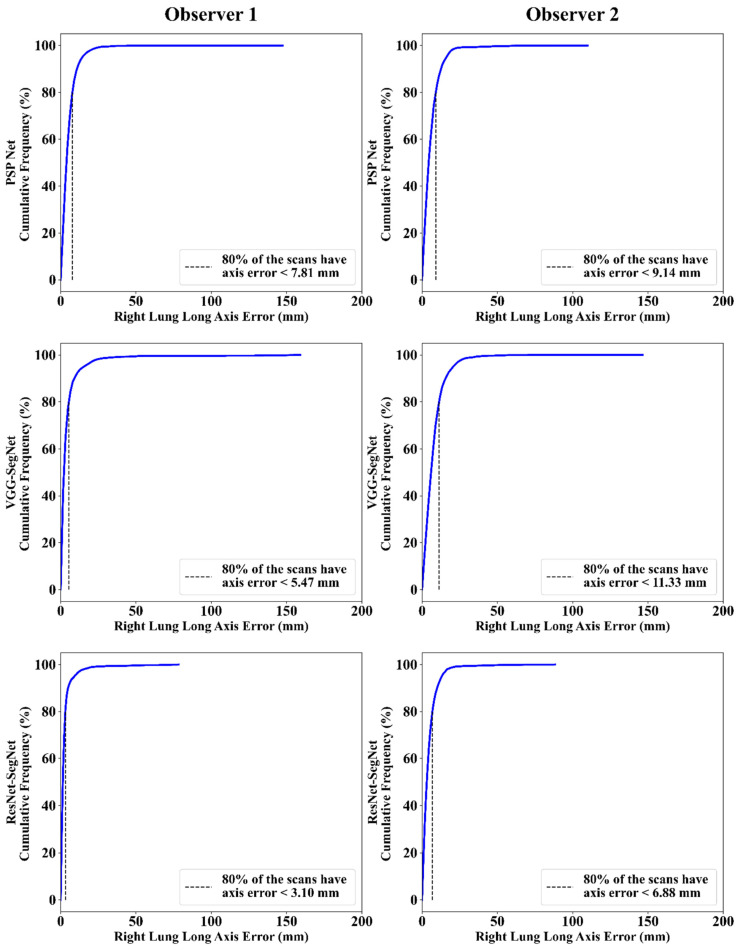
Cumulative frequency plot for right LLAE using three AI models: Observer 1 vs. Observer 2.

Figure 21 and Figure 22 show the cumulative frequency plot LLAE for left and right lung, respectively, corresponding to Observer 1 and Observer 2 for the three AI models. Based on the 80% threshold, the LLAE for the left lung (Figure 21) using the three AI models for Observer 1 and Observer 2 were 6.12 mm (for PSP Net), 4.77 mm (for VGG-SegNet), and 5.01 mm (for ResNet-SegNet) and 10.88 mm (for PSP Net), 13.30 mm (for VGG-SegNet), and 9.18 mm (for ResNet-SegNet), respectively. Similarly, for the right lung (Figure 22), the error was 7.81 mm (for PSP Net), 5.47 mm (for VGG-SegNet), and 3.10 mm (for ResNet-SegNet) and 9.14 mm (for PSP Net), 11.33 mm (for VGG-SegNet), and 6.88 mm (for ResNet-SegNet), respectively, for Observer 1 and Observer 2. The three AI models follow the order: PSP Net, VGG-SegNet, and ResNet-SegNet.

##### Correlation Plot for Lung Long Axis Error

**Figure 23:**
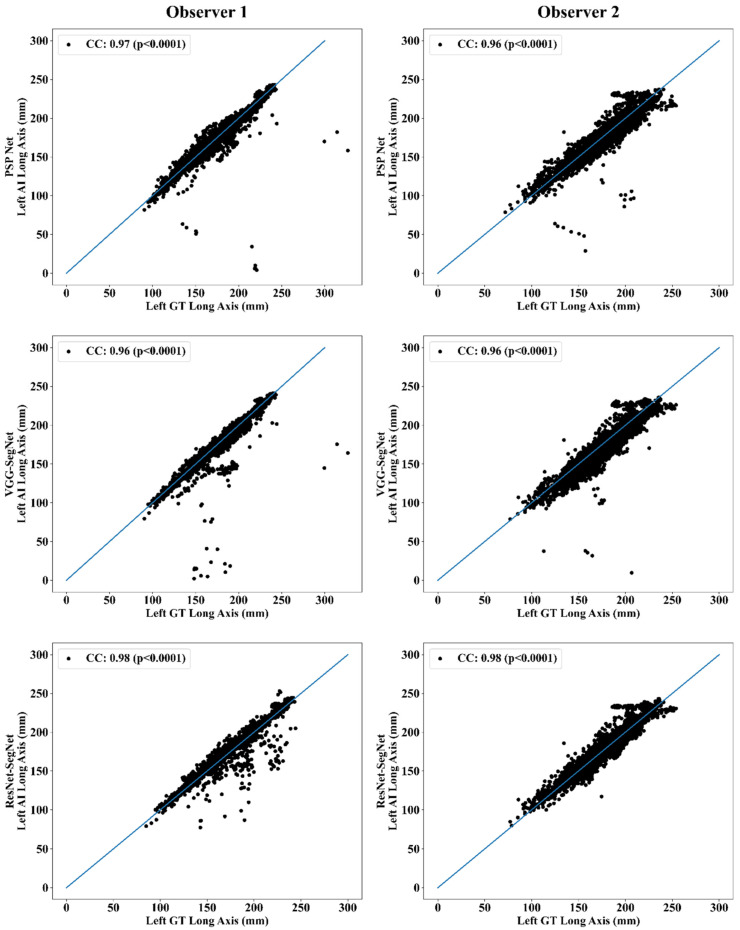
CC of left LLA for three AI models: Observer 1 vs. Observer 2.

**Figure 24:**
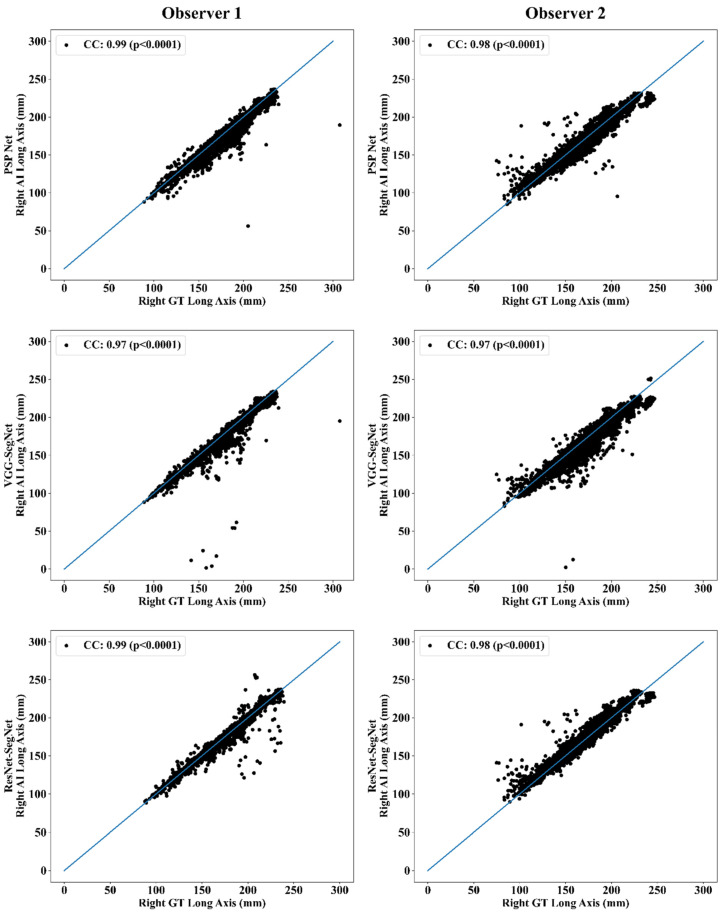
CC of right LLA using three AI models: Observer 1 vs. Observer 2.

Figure 23 and Figure 24 show the CC plot for the three AI models considered in the proposed inter-observer variability study for Observers 1 and 2. Table 2 summarizes the CC values for the left, right, and mean errors of the LLA. It proves the hypothesis that the percentage difference between the results using the two observers has a difference of <5%. This demonstrates that the proposed system is clinically valid in the suggested inter-observer variability study context.

##### Bland-Altman Plots for Lung Long Axis Error

**Figure 25:**
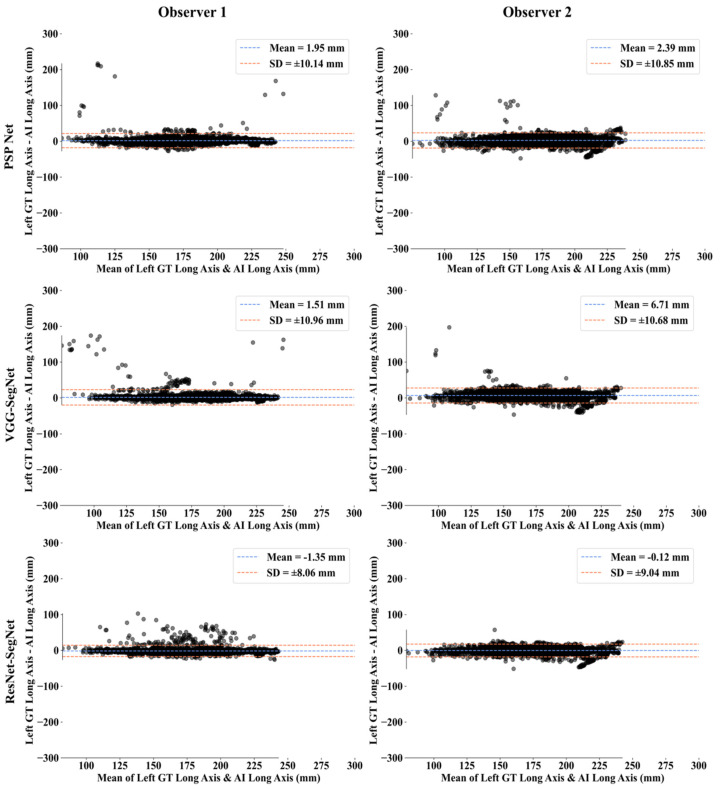
BA for the left LLA using the three: Observer 1 vs. Observer 2.

**Figure 26:**
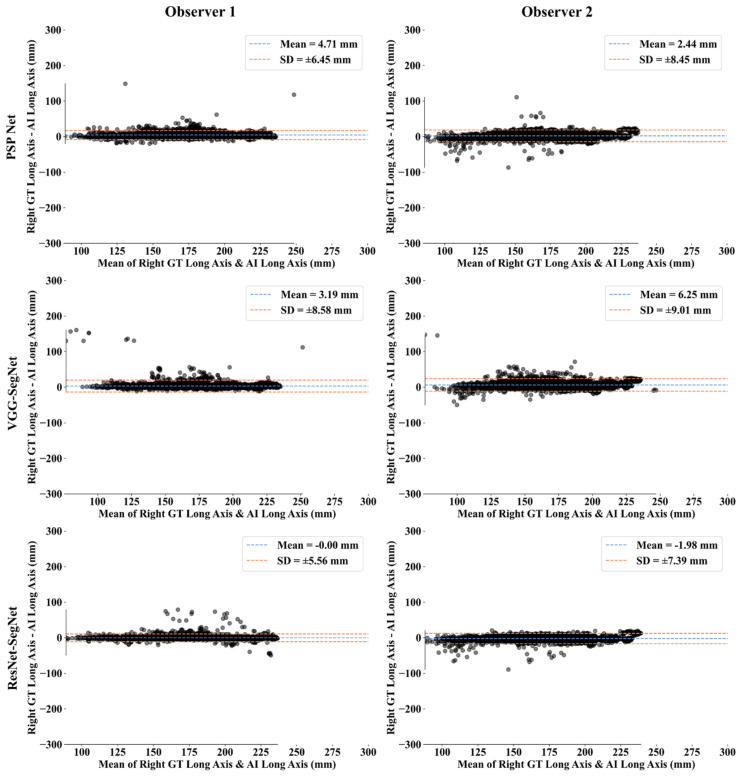
BA for the right LLA using the three AI models: Observer 1 vs. Observer 2.

The (i) mean and (ii) standard deviation of the lung long axis corresponding to Observer 1 and Observer 2 for the three AI models is shown in Figure 25 for the left lung and Figure 26 for the right lung.

##### Statistical Tests

The system’s dependability and stability were assessed using a standard paired *t*-test, ANOVA, and Wilcoxon test. The paired *t*-test can be used to see if there is enough data to support a hypothesis; the Wilcoxon test is its alternative when the distribution is not normal. ANOVA helps in the analysis of the difference between the means of groups of the input data. MedCalc software (Osteen, Belgium) was used to perform the statistical analysis. To validate the system presented in this study, we have presented all the possible combinations (twelve in total) for the three AI models between Observer 1 and Observer 2. Table 3 shows the paired *t*-test, ANOVA, and Wilcoxon test results for the 12 combinations.

##### Figure of Merit

The likelihood of the error in the system is known as the figure of merit (FoM). We have calculated FoM for (i) lung area and (ii) lung long axis to show the acceptability of the hypothesis if the % difference between the two observers is <5%. Table 4 shows the values for FoM using Equation (5) and the % difference for the three AI models against the two observers. Similarly, Table 5 shows the values for FoM using Equation (6) and the % difference for the three AI models against the two observers.

(5) $$FoM_{A}\left(m\right)=100-\left[\left(\frac{\left|{\bar{A}}_{ai}\left(m\right)-{\bar{A}}_{gt}\right|}{{\bar{A}}_{gt}}\right)\times 100\right],$$

(6) $$FoM_{LA}\left(m\right)=100-\left[\left(\frac{\left|{\bar{L}}_{ai}\left(m\right)-{\bar{L}}_{gt}\right|}{{\bar{L}}_{gt}}\right)\times 100\right]\;\mathrm{where}\;{\bar{A}}_{ai}\left(m\right)=\frac{\sum_{n\;=\;1}^{N}A_{ai}\left(m,n\right)}{N},\;{\bar{A}}_{gt}=\frac{\sum_{n\;=\;1}^{N}A_{gt}\left(n\right)}{N},\;{\bar{LA}}_{ai}\left(m\right)=\frac{\sum_{n\;=\;1}^{N}LA_{ai}\left(m,n\right)}{N}\;\&\;{\bar{LA}}_{gt}=\frac{\sum_{n\;=\;1}^{N}LA_{gt}\left(n\right)}{N}$$

## 5. Discussion

The study presented the inter-observer variability analysis for the COVLIAS 1.0 using three AI models, PSP Net, VGG-SegNet, and ResNet-SegNet. These models have considered tissue characterization approaches since they analyze the tissue data for better feature extraction to evaluate for ground vs. background, thus are more akin to a tissue characterization in classification framework [30,37]. Our group has strong experience in tissue characterization approaches with different AI models and applications for classification using ML frameworks such as plaque, liver, thyroid, breast [21,28,30,63,64,65,66,67,68], and DL framework [1,36,69,70]. These three AI models were trained using the GT annotated data from the two observers. The percentage difference between the outputs of the two AI model results was less than 5%, and thus the hypothesis was confirmed. During the training, the K5 cross-validation protocol was adapted on a set of 5000 CT images. For the PE of the proposed inter-observer variability system, the following ten metrics were considered: (i) visualization of the lung boundary, (ii) visualization of the lung long axis, cumulative frequency plots for (iii) LAE, (iv) LLAE, CC plots for (v) lung area, (vi) lung long axis, BA plots for (vii) lung area, (viii) lung long axis, (ix) ROC and AUC curve, and (x) JI and DS for estimated AI model lung regions. These matrices showed consistent and stable results. The training, evaluation, and quantification were implemented on the GPU environment (DGX V100) using python. We adapted vectorization provided by python during the implementation of the Numba library.

### 5.1. A Special Note on Three Model Behaviors with Respect to the Two OBSERVERS

The proposed inter-observer variability study used three AI models for the analysis, where PSP Net was implemented for the first time for COVID-19 lung segmentation. The other models VGG-SegNet and ResNet-SegNet were used for benchmarking. The AUC for the mean lung region for the three AI models was >0.95 for both Observer 1 and Observer 2.

Our results, shown below in Table 6, compared various metrics that included the inter-observer variability study for the three AI models. All the models behaved consistently while using the two different observers. Our results showed that ResNet-SegNet was the best performing model for all the PE metrics. The percentage difference between the two observers was 0.4%, 3.7%, and 0.4%, respectively, for the three models PSP Net, VGG-SegNet, and ResNet-SegNet, respectively. This further validated our hypothesis for every AI model, keeping the error threshold less than 5%. Even though all three AI models passed the hypothesis, VGG-SegNet is the least superior. This is because the number of the layers in the VGG-SegNet architecture (Figure 5) is 19, compared to ~50 in PSP Net (Figure 4) and 51 (encoder part) in the ResNet-SegNet model (Figure 6). By taking the results from both the observers into account, the order of the performance of the models is ResNet-SegNet > PSP Net > VGG-SegNet. Further, we also conclude that HDL models are superior to SDL (PSP Net). The aggregate score was computed as the mean for all the models for Observer 1, Observer 2, and the mean of the two Observers. Even though the performance of all the models was comparable, when carefully looking at the performance of Observer 1 the order of performance was ResNet-SegNet > VGG-SegNet > PSP Net. For Observer 2, the order of performance was ResNet-SegNet > PSP Net > VGG-SegNet. Further, the performance of the left lung was better than the right lung for the reasons unclear at this point, and more investigations would be needed to evaluate this.

### 5.2. Benchmarking

There have been several studies in the area of DL for lung segmentation, but only a few in the region of COVID-19 [71,72,73,74], and even less that involved variability analysis. Table 7 shows the benchmarking table having three variability studies: Saba et al. [48], Jeremy et al. [75], and Joskowicz et al. [76], that are compared against Suri et al. in this proposed study. Saba et al. has used a dataset of 96 patients with three observers for tracings, and ROC curves were also not presented in the study. Jeremy et al. [60] have demonstrated the variability analysis using five different observers that used the area error as the metric. The boundary error, ROC, JI, and DS were not discussed. Finally, Joskowicz et al. [76] used 480 images and 11 observers to annotate the dataset, but no area and boundary errors were present. Moreover, they did not present the ROC curves, JI, and DS for the tracings. All three studies [48,75,76], only performed manual annotation of the non-COVID dataset, and there was no involvement of the AI techniques to generate the boundaries automatically. Comparatively, the proposed study provides a first-of-its-kind for inter-observer variability analysis alongside HDL and SDL solutions, supporting our hypothesis that the error between the AI models trained using the two observers involved is less than 5%.

### 5.3. Strengths, Weakness, and Extensions

The proposed study successfully validated the hypothesis for the inter-observer variability settings, demonstrating that the difference between the two AI models when trained by the two observers was less than 5%. It was the first-time inter-observer variability was presented for COVID-19 lung segmentation using HDL and SDL models.

In spite of encouraging results, the study could not include more than two observers due to reasons such as cost, time, and availability of the radiologists. The imaging analysis component could be extended to handle more dense pulmonary opacities such as consolidation or mixed opacities during lung segmentation.

As part of the extension, the HDL models can be extended, which combines DL with ML or two solo DL models for lung segmentation. Conventional methods [77,78] can be used for lung segmentation embedded with denoising methods [79] and benchmarked against the AI models. The system can be extended to unseen data where the training data is taken from one clinical site and testing data can be from the other clinical site. It would also be interesting to explore the segmentation of lungs in the healthy patients using the AI model trained on COVID-19 patients. Other neural network techniques such as generative adversarial networks (GANs) [80] or transfer learning and loss schemes [38,44,81] can also be adapted. A big data framework can be used to integrate comorbidity factors [82] in the AI models.

## 6. Conclusions

The proposed study is the first of its kind to evaluate the effect of ground-truth tracings on the AI models for COVID-19 CT lung segmentation. Three kinds of AI models, PSP Net, VGG-SegNet, and ResNet-SegNet, were adapted for lung segmentation. Two different Observers were used to annotate 5000 CT lung slices taken from 72 COVID-19 patients. Thus, six AI training models (three AI models times two Observers) were generated and evaluated using the K5 cross-validation protocol. Ten different kinds of metrics were used for the evaluation of the six AI models. The two Observers’ error metrics were compared to validate the hypothesis for every AI model, keeping below the error threshold of 5%. Our results showed that the difference in these errors were 0%, 0.51%, and 2.04% (all < 5%), respectively, for the three AI models, validating the hypothesis. Statistical analysis was conducted using a standard paired *t*-test, ANOVA, and Wilcoxon test to prove the system’s hypothesis. The inter-variability COVLIAS 1.0 showed clinically robust and statistically stable outcomes for this pilot study and, thus, can be adapted in clinical settings.

## Figures and Tables

**Table 1:** Comparison of the CC values obtained between AI model area and the GT area corresponding to Observer 1 and Observer 2.

	PSP Net			VGG-SegNet			ResNet-SegNet		
	Left	Right	Mean	Left	Right	Mean	Left	Right	Mean
**Observer 1**	0.98	0.98	0.98	0.98	0.99	0.99	0.98	0.98	0.98
**Observer 2**	0.98	0.98	0.98	0.98	0.98	0.98	1.00	1.00	1.00
**% Difference**	0.00	0.00	0.00	0.00	1.01	0.51	2.04	2.04	2.04

**Table 2:** Comparison of the CC values obtained between AI model lung long axis and the GT lung long axis corresponding to Observer 1 and Observer 2.

	PSP Net			VGG-SegNet			ResNet-SegNet		
	Left	Right	Mean	Left	Right	Mean	Left	Right	Mean
**Observer 1**	0.97	0.99	0.98	0.96	0.97	0.97	0.98	0.99	0.99
**Observer 2**	0.96	0.98	0.97	0.96	0.97	0.97	0.98	0.98	0.98
**% Difference**	1.03	1.01	1.02	0.00	0.00	0.00	0.00	1.01	0.51

**Table 3:** Paired *t*-test, Wilcoxon, ANOVA, and CC for LA and LLA for the 12 combinations.

		Lung Area				Lung Long Axis			
*SN*	Combinations	Paired *t*-Test (*p*-Value)	Wilcoxon (*p*-Value)	ANOVA (*p*-Value)	CC [0–1]	Paired *t*-Test (*p*-Value)	Wilcoxon (*p*-Value)	ANOVA (*p*-Value)	CC [0–1]
1	P1 vs. V1	<0.0001	<0.0001	<0.001	0.9726	<0.0001	<0.0001	<0.001	0.9509
2	P1 vs. R1	<0.0001	<0.0001	<0.001	0.9514	<0.0001	<0.0001	<0.001	0.9506
3	P1 vs. P2	<0.0001	<0.0001	<0.001	0.9703	<0.0001	<0.0001	<0.001	0.9686
4	P1 vs. V2	<0.0001	<0.0001	<0.001	0.9446	<0.0001	<0.0001	<0.001	0.9445
5	P1 vs. R2	<0.0001	<0.0001	<0.001	0.9764	<0.0001	<0.0001	<0.001	0.9661
6	V1 vs. R1	<0.0001	<0.0001	<0.001	0.9663	<0.0001	<0.0001	<0.001	0.9561
7	V1 vs. P2	<0.0001	<0.0001	<0.001	0.9726	<0.0001	<0.0001	<0.001	0.9671
8	V1 vs. V2	<0.0001	<0.0001	<0.001	0.9766	<0.0001	<0.0001	<0.001	0.9638
9	V1 vs. R2	<0.0001	<0.0001	<0.001	0.9943	<0.0001	<0.0001	<0.001	0.9796
10	R1 vs. P2	<0.0001	<0.0001	<0.001	0.9549	<0.0001	<0.0001	<0.001	0.9617
11	R1 vs. V2	<0.0001	<0.0001	<0.001	0.9513	<0.0001	<0.0001	<0.001	0.9499
12	R1 vs. R2	<0.0001	<0.0001	<0.001	0.9690	<0.0001	<0.0001	<0.001	0.9726

CC: Correlation coefficient; P1: PSP Net for Observer 1; V1: VGG-SegNet for Observer 1; R1: ResNet-SegNet for Observer 1; P2: PSP Net for Observer 2; V2: VGG-SegNet for Observer 2; R2: ResNet-SegNet for Observer 2.

**Table 4:** FoM for lung area.

	Observer 1			Observer 2			% Difference			Hypothesis (<5%)		
	Left	Right	Mean	Left	Right	Mean	Left	Right	Mean	Left	Right	Mean
**PSP Net**	95.07	95.11	95.09	97.37	97.49	97.43	2%	3%	2%	✓	✓	✓
**VGG-SegNet**	96.73	97.40	97.04	97.74	97.27	97.52	1%	0%	0%	✓	✓	✓
**ResNet-SegNet**	98.33	99.98	99.11	97.88	99.20	98.50	0%	1%	1%	✓	✓	✓

**Table 5:** FoM for lung long axis.

	Observer 1			Observer 2			% Difference			Hypothesis (<5%)		
	Left	Right	Mean	Left	Right	Mean	Left	Right	Mean	Left	Right	Mean
**PSP Net**	98.91	97.34	98.13	98.65	98.60	98.62	0%	1%	1%	✓	✓	✓
**VGG-SegNet**	99.41	98.50	98.95	97.07	97.27	97.17	2%	1%	2%	✓	✓	✓
**ResNet-SegNet**	99.73	99.37	99.83	99.51	98.75	99.13	0%	1%	1%	✓	✓	✓

**Table 6:** Comparison of PE metrics for Observer 1 and Observer 2 and their mean.

	Observer 1			Observer 2			Mean Obs. 1 & Obs. 2		
Attributes	PSP Net	VGG-SegNet	ResNet-SegNet	PSP Net	VGG-SegNet	ResNet-SegNet	PSP Net	VGG-SegNet	ResNet-SegNet
DS	0.96	0.98	0.98	0.96	0.95	0.97	0.96	0.97	0.98
JI	0.93	0.96	0.97	0.92	0.9	0.94	0.93	0.93	0.96
CC Left LA	0.98	0.98	0.98	0.98	0.98	1	0.98	0.98	0.99
CC Right LA	0.98	0.99	0.98	0.98	0.98	1	0.98	0.99	0.99
CC Left LLA	0.97	0.96	0.98	0.96	0.96	0.98	0.97	0.96	0.98
CC Right LLA	0.99	0.97	0.99	0.98	0.97	0.98	0.99	0.97	0.99
CF Left LA < 10%	0.83	0.85	0.90	0.81	0.75	0.89	0.82	0.80	0.89
CF Right LA < 10%	0.78	0.85	0.90	0.80	0.75	0.88	0.79	0.80	0.89
**Aggregate Score**	**7.42**	**7.54**	**7.67**	**7.39**	**7.24**	**7.64**	**7.40**	**7.39**	**7.66**

DS: Dice similarity; JI: Jaccard index; CC: Correlation coefficient; LA: Lung area; LLA: Lung long axis; CF: Cumulative frequency; Obs: Observer.

**Table 7:** Benchmarking Table.

Attributes/Author	Saba et al. [49]	Jeremy et al. [77]	Joskowicz et al. [78]	Suri et al. (Proposed)
# of patients	96	33	18	72
# of Images	NA	NA	490	5000
# of Observers	3	5	11	2
Dataset	Non-COVID	Non-COVID	Non-COVID	COVID
Image Size	512	NA	512	768
# of tests/PE	5	0	2	13
CC	0.98	NA	NA	0.98
Boundary estimation	Manual	Manual	Manual	Manual & automatic
AI Models	NA	NA	NA	3
Modality	CT	CT	CT	CT
Area Error	✓	✓	✗	✓
Boundary Error	✓	✗	✗	✓
ROC	✗	✗	✗	✓
JI	✓	✗	✗	✓
DS	✓	✗	✗	✓

CC: Correlation coefficient; ROC: Receiver-Operating Characteristics; DS: Dice similarity; JI: Jaccard index.

## Data Availability

Not applicable.

## Abbreviations

**SN**	**Symbol**	**Description of the Symbols**
1	ACC (ai)	Accuracy
2	AE	Area Error
3	AI	Artificial Intelligence
4	ARDS	Acute Respiratory Distress Syndrome
5	AUC	Area Under the Curve
6	BA	Bland-Altman
7	BE	Boundary Error
8	CC	Correlation coefficient
9	CE	Cross Entropy
10	COVID	Coronavirus disease
11	COVLIAS	COVID Lung Image Analysis System
12	CT	Computed Tomography
13	DL	Deep Learning
14	DS	Dice Similarity
15	FoM	Figure of merit
16	GT	Ground Truth
17	HDL	Hybrid Deep Learning
18	IS	Image Size
19	JI	Jaccard Index
20	LAE	Lung Area Error
21	LLAE	Lung Long Axis Error
22	NIH	National Institute of Health
23	PC	Pixel Counting
24	RF	Resolution Factor
25	ROC	Receiver operating characteristic
26	SDL	Solo Deep Learning
27	VGG	Visual Geometric Group
28	VS	Variability studies
29	WHO	World Health Organization

## Symbols

**SN**	**Symbol**	**Description of the Symbols**
1	$l_{CE}$	Cross Entropy-loss
2	m	Model used for segmentation in the total number of models M
3	n	Image scan number in total number *N*
4	${\bar{A}}_{ai}\left(m\right)$	Mean estimated lung area for all images using AI model ‘*m*’
5	$A_{ai}\left(m,n\right)$	Estimated Lung Area using AI model ‘m’ and image ‘*n*’
6	$A_{gt}\left(n\right)$	GT lung area for image ‘*n*’
7	${\bar{A}}_{gt}$	Mean ground truth area for all images N in the database
8	${\bar{LA}}_{ai}\left(m\right)$	Mean estimated lung long axis for all images using AI model ‘*m*’
9	$LA_{ai}\left(m,n\right)$	Estimated lung long axis using AI model ‘m’ and image ‘*n*’
10	$LA_{gt}\left(n\right)$	GT lung long axis for image ‘*n*’
11	${\bar{LA}}_{gt}$	Mean ground truth long axis for all images N in the database
12	$FoM_{A}\left(m\right)$	Figure-of-Merit for segmentation model ‘*m*’
14	$FoM_{LA}\left(m\right)$	Figure-of-Merit for long axis for model ‘*m*’
15	JI	Jaccard Index for a specific segmentation model
16	DSC	Dice Similarity Coefficient for a specific segmentation model
17	TP, TN	True Positive and True Negative
18	FP, FN	False Positive and False Negative
19	*x*_i_	GT label
20	p_i_	SoftMax classifier probability
21	*Y_p_*	Ground truth image
22	$\hat{Y_{p}}$	Estimated image
23	*P*	Total no of pixels in an image in *x, y*-direction
24	K5	Cross-validation protocol with 80% training and 20% testing (5 folds)
**Deep Learning Segmentation Architectures**		
25	PSP Net	SDL model for lung segmentation with pyramidal feature extraction
26	VGG-SegNet	HDL model designed by fusion of VGG-19 and SegNet architecture
27	ResNet-SegNet	HDL model designed by fusion of ResNet-50 and SegNet architecture
